# Food-Grade Pickering Emulsions: Preparation, Stabilization and Applications

**DOI:** 10.3390/molecules25143202

**Published:** 2020-07-14

**Authors:** Lijuan Chen, Fen Ao, Xuemei Ge, Wen Shen

**Affiliations:** 1 Department of Food Science and Technology, College of Light Industry Science and Engineering, Nanjing Forestry University, Nanjing 210037, China; 13813972326@163.com; 2 School of Food and Biological Engineering, Shaanxi University of Science & Technology, Xi’an 710000, China; 18309292650@163.com

**Keywords:** Pickering emulsion, solid particles, stabilization mechanism, food applications

## Abstract

In recent years, Pickering emulsions have emerged as a new method and have attracted much attention in the fields of food sciences. Unlike conventional emulsions, Pickering emulsions are stabilized by solid particles, which can irreversibly adsorb on the oil-water interface to form a dense film to prevent the aggregation of droplets. The research and development of food-grade solid particles are increasingly favored by scientific researchers. Compared with conventional emulsions, Pickering emulsions have many advantages, such as fewer using amounts of emulsifiers, biocompatibility and higher safety, which may offer feasibility to have broad application prospects in a wide range of fields. In this article, we review the preparation methods, stabilization mechanism, degradation of Pickering emulsions. We also summarize its applications in food sciences in recent years and discuss its future prospects and challenges in this work.

## 1. Introduction

Emulsions are a dispersion system composed of two incompatible liquids, that is, a mixture of one liquid in the form of small droplets dispersed in another liquid. Emulsions are ordinarily composed of water and oil and it is customary to refer to organic liquids that are insoluble in water as oil phase. Since the emulsion is a thermodynamically unstable system, an emulsifier is essentially required to achieve desired stability. Conventional emulsifiers are generally small molecule surfactants which mainly include ionic surfactants and non-ionic surfactants and amphiphilic biopolymers. However, some of these emulsifiers are harmful to human health, so applications are limited in food, cosmetics, and pharmaceutical industries.

Pickering emulsion is an emulsion which was stabilized by solid particles as an emulsifier. This phenomenon was first discovered by Ramsden in 1903, but first described in literature by Pickering in 1907. It is reported that emulsification of emulsions relied on the size of solid particles, which could be adsorbed on the globules to form a coating to prevent them from gathering [1]. Solid particles have certain wettability in both aqueous and oil phases but are insoluble in any phase [2], so they can irreversibly adsorb and fix on the oil-water interface and then around the droplets to form a dense adsorption layer, which provides a physical barrier in space to prevent the contact between the droplets. In addition, different wetting properties of particles in two phases are represented by three-phase contact angle θ, which can determine the types of Pickering emulsions. By adding as-prepared solid particles suspension to the emulsion, a Pickering emulsion can be prepared with emulsification techniques such as high-speed homogenization, high-pressure homogenization, and ultrasound.

Compared with conventional emulsions, Pickering emulsions have many unique advantages as follows: (i) Pickering emulsions use solid particles as emulsifiers to stabilize the emulsion, which can be irreversibly adsorbed at the oil-water interface to form a film to prevent the aggregation of oil droplets. (ii) Pickering emulsion is not susceptible to external environment, such as changing the pH, ionic strength, temperature, and oil phase composition of the system. (iii) Compared to emulsions stabilized with conventional surfactants, Pickering emulsions require fewer amounts of stabilizers during the preparation process. (iv) In addition, since solid particulate materials used as stabilizers are mostly edible natural substances, Pickering emulsions have good biocompatibility and can be used as carriers for the delivery of bioactive substances. Solid particles commonly used to stabilize Pickering emulsions are: food-grade organic materials such as polysaccharide particles, protein particles, lipid particles, and safe inorganic particles such as silica, calcium carbonate, and hydroxyapatite (Hap) [3]. Therefore, Pickering emulsion became a very important method and was widely used in food products processing in recent years. With the increase in food safety and health requirements, researchers gradually began to focus on the use of food-grade solid particles to stabilize the Pickering emulsions. They use natural proteins, polysaccharides, polyphenols, etc. as raw materials to obtain solid particles through modification, compounding and other control methods and to improve the stability of Pickering emulsions. However, the stability of Pickering emulsions prepared from food-grade materials is not very high, and the cost is expensive. Besides, it is difficult to achieve large-scale industrial production. These problems are urgently needed to be solved in this field. With the development of food emulsion, more rationales of the Pickering stabilized systems were designed and optimized to be applied in food. Some current trends such as how to improve the nutrient bioavailability during food digestion, nanoparticles used as emulsifying agents and Pickering stabilizers were introduced in recent reported reviews [4,5,6]. In this work, we mainly introduce the structure and composition of Pickering emulsion, which involving the mechanism of materials in formulating Pickering emulsion and some particles potentially to be used in food-degrade Pickering emulsions such as Janus was also described. Preparation, methods for stabilizing Pickering emulsions and their recent applications were summarized in this review.

## 2. Structure and Composition of Pickering Emulsions

### 2.1. Oil Phase and Aqueous Phase

**Figure 1:**
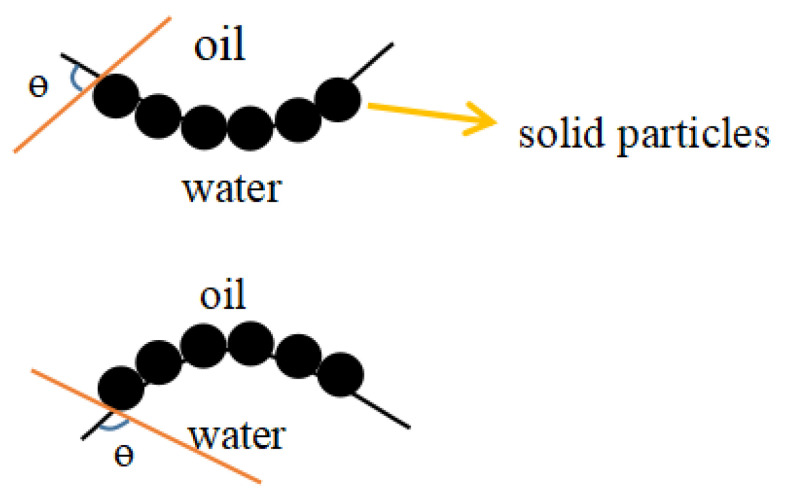
Description of two types of Pickering emulsions.

The droplets of one liquid phase dispersed into the other phase through mechanical agitation and adding emulsifiers are called emulsions, which can form either oil-in-water (O/W) or water-in-oil (W/O) emulsions [7]. Two types of Pickering emulsions are shown in Figure 1. Among them, the phase forming droplets is called a dispersed phase, and the phase where droplets are dispersed to prevent aggregation is called a continuous phase [7]. Therefore, the important components of the Pickering emulsion are the oil phase and the water phase, which are incompatible with each other. It is customary to refer to water or water solution as the aqueous phase, and water-insoluble organic liquids such as benzene and kerosene are collectively referred to as the oil phase. The two phases can form an unstable dispersion system by mechanical stirring, which requires an emulsifier to maintain stability.

### 2.2. Solid Particles

Recently, the insecurity of surfactant molecules in conventional emulsifiers has caused more and more concerns [7,8,9]. Solid micro or nanoparticles which replace conventional surfactant, as the emulsifier of the emulsion, play a key role in the preparation of Pickering emulsions [10,11]. These solid particles must meet certain conditions to act as a stabilizer for Pickering emulsions: First of all, the particles should have a certain degree of wettability, which make them have sufficient interface adsorption efficiency, in both phases but insoluble in either phase. Secondly, the size of particles should be smaller than the emulsion droplet at least an order of magnitude. In addition, the concentration, wettability and surface charge of solid particles will affect the stability of Pickering emulsion. The solid particles commonly used to stabilize Pickering emulsions are polysaccharide particles, protein particles, lipid particles and inorganic particles [12,13,14,15,16].

#### 2.2.1. Types of Solid Particles

##### Starch Particles

Starch is a high molecular weight polysaccharide composed of a single type of sugar unit, which has several advantages such as economical, biocompatible, biodegradable and non-toxic [2]. Native starch granules have potential as food-grade Pickering stabilizers due to their morphology (size, shape) and composition [2]. However, native starch particles have the disadvantages of poor stability, poor hydrophobicity and large particle size, the effect on the emulsification of Pickering emulsions is poor, which cannot be well adsorbed at the oil–water interface.

Apparently, using different modifications, including acid hydrolysis, nanoprecipitation, enzymolysis and recrystallization methods can increase hydrophobicity of starch particle, which increases the stability of Pickering emulsions [17,18,19]. Song et al. prepared Pickering emulsions with rice starch, waxy corn starch, wheat starch, and potato starch, which were esterified by octenyl succinic anhydride [20]. Studies showed that the emulsion stabilized with rice starch particles had the minimum droplet size and good physical stability. Therefore, considering the poor hydrophobicity of starch granules, modification is necessary to more successfully stabilize O/W Pickering emulsions [3,21]. Natural starch has strong hydrophilicity. Generally, octenyl succinic anhydride (OSA) can be added to increase the hydrophobicity of starch. Song et al. used octenyl succinic anhydride (OSA) to modify indica rice starch as particle stabilizers which were used to prepare Soybean oil-in-water (O/W) Pickering emulsions [22]. Ge et al. used a nanoprecipitation method preparing corn, tapioca, and sweet potato SNPs. Herein, the three nanoparticles, which had nearly neutral wettability (θ_ow_∼90°), could promote adsorption at the oil–water interface and thus formed a suitable Pickering emulsion [23]. In the research work of Ahmad et al., they used an acid hydrolysis method obtaining round and oval-shaped Sago starch nanocrystals (Sago-SNC), which could prepare the Pickering emulsion with good stability [19]. In addition, Ke et al. prepared Pickering emulsion using starch nanocrystals with alkaline treatment to decrease the particle sizes and thus to improve the stability [21].

##### Chitosan

Chitosan, found in shrimp shells, crab shells and fungi, is the product of deacetylation of chitin and the only biodegradable polymer material with cations that exists in nature. Chitosan, often used as a drug delivery and film-forming material, is a polysaccharide which was proven to be safe, non-toxic, antibacterial, and has good biodegradability [24,25,26]. Chitosan, with the free amino and hydroxyl groups along its backbone which makes it very useful in biomedicine and pharmaceutics, are widely used due to its biodegradability and biocompatibility [3].

However, owing to the hydrophilic amine (-NH3^+^) and the hydroxide (-OH^−^) groups embedded along its backbone, chitosan is not a good emulsifier by nature, which possesses low surface activity. Considering some experimental results observed, it is obvious that the hydrophobicity of chitosan can be changed by adjusting pH and the degree of deacetylation. In a low pH solution, the protonation of the amino-group on the molecule makes the molecular chain positively charged, which can provide a strong electrostatic repulsion, thereby improving the stability of the emulsion. However, under neutral and basic environments, chitosan molecules lead to precipitation and limited application in Pickering emulsions, because the charge of them were absent near their pKa of 6.5. Therefore, chitosan hydrochloride (CHC), an important water-soluble chitosan derivative, was chose to obtain nanoparticles [27]. In addition, research showed optimal emulsification with moderate deacetylation. In the research work by Ho et al., comparing self-aggregated chitosan particles prepared with and without ultrasonication pretreatment [28]. It indicated that the emulsification effect of chitosan before ultrasonication was greater than that after ultrasonication. This is because ultrasonic treatment reduced the hydrophobicity of chitosan. Additionally, because chitosan has the positively charged amino groups, through the ionic cross-linking method, composite particles formed by interaction with a negatively charged polyanion group, such as sodium tripolyphosphate, can also be used as Pickering emulsifier. In the research of Tian et al., they investigated the influence of the contents of chitosan-tripolyphosphate particles (CS-TPP Ps) on the physical and chemical properties of the Pickering emulsions at 40 °C for 14 days. So far as can be seen, the citral Pickering emulsions became a little yellow after the storage of 14 days at 40 °C, due to the oxidation of citral, and howbeit this cannot affect the emulsifying properties of CS-TPP Ps [29]. On the other hand, three types of gliadin–chitosan nanoparticles (GCNPs), including primary complexation, soluble complexes, and coacervates, were used in the research of Li et al., which could stabilize Pickering emulsion. The result shows that the coacervate-stabilized Pickering emulsion had the highest viscoelasticity and solid-like behavior [30].

##### Cellulose Microparticles or Nanocrystals

Cellulose, as one of most widely distributed and abundant material in nature, is with macromolecular polysaccharide composed of glucose [31,32]. It is insoluble in water and general organic solvents. It is the main structural component of plant cell walls and biofilms produced by certain bacteria. It has been demonstrated that cotton’s cellulose content is close to 100%, which is the pure natural cellulose source and wood’s cellulose accounts for 40–50%. Cellulose and its derivatives when the hydroxyl groups in cellulose are replaced by methyl, hydroxypropylmethyl and carboxymethyl can be used as Pickering particles to prepare emulsions. To obtain cellulose product in Pickering emulsion, chemical and mechanical methods are be used. Native cellulose exists as macroscopic fibers or as microfibrillated cellulose (MFC) and microcrystalline cellulose (MCC) and nanocrystalline cellulose (NCC) can be prepared by acid hydrolysis [2].

In the research of Sanchez-Salvador et al., O/W Pickering emulsions could be obtained by using cellulose microfibers (CMF) produced from cotton cellulose linters by mechanical treatment through a high-pressure homogenizer [33]. Results showed that the apparent viscosity of the emulsion phase in Pickering emulsion containing 1.0 wt% CMF increase 60–90 times with respect to the sunflower oil. In addition, Yokota et al. reported that cellulose nanofibrils prepared by aqueous counter collision had higher emulsification abilities due to exposure of inherently hydrophobic surface planes of it [34]. In the research work of Gong et al., modified oxidized cellulose nanocrystals (m-O-CNCs) were used as Pickering emulsion stabilizer due to hydrophobic domains comprised of phenyl groups [35]. Additionally, Zhai et al. used bacterial cellulose (BC) nanoparticles with the sustainability and good bio-compatibility by hydrochloric acid hydrolysis to stabilize Pickering emulsions with a peanut oil concentration of 15% (*v*/*v*) [36]. Herein, BC nanoparticles had good hydrophilic and lipophilic properties and the oil-in-water emulsion had the good stability with only 0.05% (*w*/*v*) BC nanofibers at neutral pH. Therefore, the emulsion stabilized by low-concentration nanofibers can be used as a new food-grade Pickering emulsion, which has great potential in the food industry.

##### Whey Protein

Whey protein from animal origin is one kind of food-grade material, which can be applied to food formulations, such as ice cream, soft cheese, and semi-hard cheese, due to a variety of nutritional values and functionalities [37,38]. Nevertheless, due to the susceptibility of proteins in heat treatment, which is the necessary during food procedure, whey protein appears denature, causing instability of the emulsion. To iron out this problem of unsteadiness, oceans of methods of processing of whey protein have been developed by massive researchers [39]. It was proposed by Liu et al. that whey protein heat-denatured aggregates could be used to make Pickering emulsions. It has been extensively studied that whey protein isolate, which is a protein obtained after further extraction on the basis of concentrated whey protein, could be used as emulsifiers after adding tannic acid. Besides, Liu et al. reported that nanofibrils produced from whey protein isolate (WPI) glycated with glucose, lactose, or maltodextrin by heating at pH 2.0 and 90 °C could form and stabilize O/W Pickering emulsions [38]. Herein, glycation changes the surface hydrophobicity and charge properties of proteins facilitating adsorption and aggregation of fibrils on oil droplets and providing emulsion stability. Additionally, Yi et al. prepared β-Carotene-loaded high internal phase Pickering emulsions with spherical whey protein isolate nanoparticles based on Ca^2+^ induced cross-linking [14]. Lv et al. reported that whey protein isolate (WPI) gel particles could be prepared via high hydrostatic pressure (HHP) treatment, which is a novel non-thermal and environmentally friendly technology with little effects on the quality of foods. In the research, WPI gel particles were used as stabilizers to fabricate the curcumin-loaded Pickering emulsions with desired stability [40].

##### Zein

Zein extracted from corn, a protein with the high percentage of nonpolar amino acids (leucine, alanine, and proline) and a sub-product of the food and ethanol industry, is a natural amphiphilic polymer material, which has been extensively studied in biogels, foods, cosmetics and pharmaceutical industry [5,41,42,43]. As a kind of water-insoluble food-grade alcohol-soluble protein, zein can form colloidal particles through self-assembly without surface-modification when pH deviated from zein isoelectric point [44]. Besides, due to the low solubility of it in both water and oil, zein colloidal particles can be fabricated through anti-solvent precipitation. However, stability of zein-stabilized emulsions was affected by particle concentration, pH and ionic strength.

Apparently, studies have been renewed in recent years, oceans of researches reported that composite particles with higher stability could be created using biodegradable and edible materials, such as chitosan, sodium caseinate, and sodium alginate, via electrostatic adsorption [44]. In the research of J. Santos et al., food-grade sunflower O/W Pickering emulsions stabilized by xanthan gum–zein complex, which had great emulsifying and stabilizing properties, were prepared [45]. Herein, advance performance xanthan gum was incorporated causing the occurrence of viscoelastic properties and a clear increase in zero-shear viscosity. In addition, the protein-polysaccharide complex formed a layer covering the droplets enhancing the stability of these emulsions. Zhu et al. prepared zein/corn fiber gum (CFG) complex particles (ZCPs) via anti-solvent precipitation method, and the presence of CFG could change the wetting properties of zein particles via electrostatic deposition. When the mass ratio of zein to CFG was 2:1, ZCPs obtained the intermediate wettability with a contact angle close to 90°, and the Pickering emulsion exhibited better stability [41].

##### Soy Protein

Soy protein is a plant-based protein containing nine essential amino acids, flavonoids, vitamin E, and other active ingredients [46,47]. It can be equivalent to animal protein due to nutritional value, and the closest to human amino acid in genetic structure. Additionally, soy isoflavones, a unique physiologically active substance, has a cholesterol-lowering function. Some studies have reported that glycinin and β-conglycinin, the two major components of soy protein, show peculiar behavior of globular molecules consisting of a hydrophilic shell and a hydrophobic kernel in aqueous environment [5]. Therefore, soy protein particles have good emulsifying and gelling properties without additional chemical modification [48]. However, in terms of external conditions, including pH, ionic strength or temperature, the solubility and stabilizing behavior of soy protein in Pickering emulsions will be affected. In the research of Wang et al., a soy protein isolate/pectin binary complex particle with electrostatic interaction generated an environmental friendliness and emulsion containing quercetin with improved stability embedded by ultrasound treatment [49]. It was proposed that emulsion at pH 3.0 exhibited good freeze-thaw stability and viscoelasticity stability. Besides, Tolulope et al. decorated okara dietary fiber (ODF) micronized with soy protein isolate (SPI) through a Maillard reaction by dry heating at 60 °C forming ODF-SPI conjugates, which presented Pickering emulsion with fabulous stability [50].

##### Fat Crystals

Based on oceans experiments, we have found that fat crystals were used as emulsifiers to stabilize water-in-oil (W/O) emulsions, with the aqueous phase dispersed as droplets in the continuous oil phase [51,52]. In the research of Supratim et al., three types of Pickering stabilization were reported [52]. Firstly, if fat crystals are surface-inactive, such as triglycerides, emulsion can be stabilized by network stabilization mechanism, which physically encases dispersed phase in the continuous crystalline matrix forming a fat crystal network by van der Waals interactions [52]. Besides, when fat crystals are amphiphilic and surface-active, they can form crystalline monolayers adsorbing onto the oil-water interface, which provides a steric barrier to coalescence and emulsion stabilization. Finally, in some cases, fat crystals can stabilize emulsions by combined Pickering and network stabilization. Wang et al. used fat crystals and nonionic surfactants to stabilize a non-aqueous self-double-emulsifying delivery system. In the study, primary O/O emulsions were prepared by mixing the inner oil phase (rutin-containing glycerol), the outer oil phase, fat crystals (glycerol monostearate, GMS) and polyglycerol polyricinoleate (PGPR) together. And then when diluted with distilled water, the rutin-loaded SDEDS could transform into O/O/W double emulsions. Herein, a mixture of 12% GMS crystals and 12% PGPR could improve the stability of the emulsions [53].

##### Hydroxyapatite

Hydroxyapatite (HAp, Ca_10_(PO_4_)_6_(OH)_2_), a tooth or bone inducer, is a surface-active material which has similar chemical composition and structure with the hard tissues of living organisms. Hydroxyapatite has no stimulation and rejection effect on tissues after being implanted into the human body due to the good biological activity and biocompatibility and now widely used in the field of biomedicine [54,55,56]. Oceans of researches demonstrated that HAp could be used as a stabilizer to fabricate Pickering emulsions. On one hand, using O/W Pickering emulsions stabilized by unmodified HAp nanoparticles could obtain microspheres including HAp nanoparticles coated PCL, PLLA, and PLGA microspheres. On the other hand, porous materials, such as HAp/PLLA porous scaffolds, HAp/PLGA porous scaffolds, were fabricated employing W/O Pickering emulsion stabilized by modified HAp nanoparticles [55]. Additionally, Pickering emulsions prepared based on HAp nanoparticles also has good physical stability and rheological properties, indicating that it has good spreading properties, which shows good application prospects in the field of food and cosmetics. Zhang et al. indicated that the interaction between PLLA and HAp could promote HAp particles adsorbed on oil–water interface. Song et al. used hydroxyapatite nanoparticles/PLLA as emulsifier, and composites nonionic surfactant sorbitan monooleate (Span 80) was used to adjust the connectivity of the pores. In the research, with an increase in Span 80 concentration from 0 to 20 mM, emulsion undergo a phase inversion from oil-in-water (O/W) type to water-in-oil (W/O) type due to the interaction of forming hydrogen bonds between Span 80 and Hap [57]. Additionally, HAp nanoparticles became the main emulsifier adsorbing at the emulsion droplet surfaces at low Span 80 concentration (5 mM), forming O/W emulsions. At higher Span 80 concentration (from 10 to 20 mM), its wettability is greatly changed and the lower adsorption energy of HAp caused the emulsion stability to decrease.

### 2.3. Janus Particles

**Figure 2:**
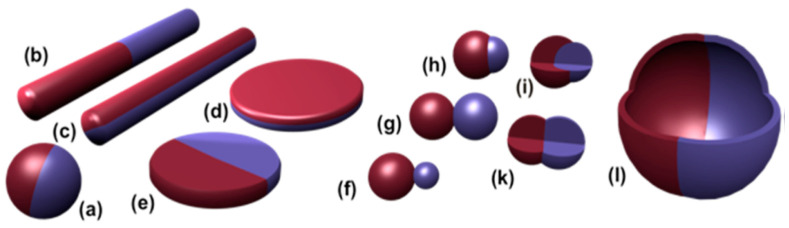
Description of Janus particles in different shapes. (**a**) Spherical. (**b**,**c**) Two different types of cylindrical. (**d**,**e**) Two different types of disc-shaped Janus. (**f**) Snowman character. (**g**,**k**) Symmetric appearance. (**h**) Attached nodes. (**i**) Eccentric encapsulation. (**l**) Janus vesicles or capsules [60].

Janus particles, which consist different chemical or physical components and structure in one single particles, have attracted extensive interests since the concept was raised in 1991. The scheme description of different types of Janus was as shown in Figure 2, such as spherical, cyclindrical or disc in sharps [58,59,60]. The Janus, which was formed by chitosan and silver eco-friendly, was reported to be applied in antimicrobial activity of B. cinerea. This could be used as advanced functional material candidate in food industry to enhancing the quality of products [61]. The amphiphilic structure of these particles may offer a feasibility to be used as surfactants due to their structure with hydrophobic domain facing oil and hydrophilic domain facing water phase [62]. Pickering emulsions using of homogeneous wettability solid particles were not thermodynamically stable. The Janus particles in Pickering emulsion could provide a feasibility to avoid formed with homogeneous wettability of spherical solid particles [63]. The Janus particles could be used as emulsifiers to stabilize Pickering emulsions [64]. It is found that the particle contact angle at oil/water interface could be tuned by optimizing size ratio of hydrophilic/hydrophobic domain. The Pickering emulsion could be stabilized by using Janus particles with proper particle architecture. Janus particles with controlled size (128 to 440 μm, hydrophilic/hydrophobic volume ratio 0.36 to 12.77) was reported to be prepared by glass capillary microfluidics. Stability determined by centrifuge shown that the emulsion drop diameter using particle with smallest size was not increased significantly at the relative centrifugal force from 0 to 150× *g*, which may indicate that the stability was relative increased [65]. The application of the Janus particles in food industry could be developed further with more finding in this area [66].

## 3. Preparation Methods of Pickering Emulsions

### 3.1. Preparation of Solid Particles

Obviously, solid particles, stabilizers for Pickering emulsions owing to having a certain degree of adsorption in both phases, play a vital role in the preparation of Pickering emulsions [8]. The solid particle size of the Pickering emulsion is usually at least in an order of magnitude smaller than the emulsion particle size. In addition, the solid particle concentration, surface charge, and wettability can also affect the stability of Pickering emulsions. In summary, the sizes, properties, and preparation methods are different due to the diversity of solid particles used in preparing Pickering emulsions.

#### 3.1.1. Mechanical or Chemical Breakdown Methods

Safety of food has become a big concern in recent years, the research on food-grade solid particles in Pickering emulsion has become more important to be investigated in-depth. Food-grade solid particles are mostly natural organisms with large sizes, such as natural starch and cellulose, their size can be reduced through mechanical or chemical methods. Commonly used mechanical methods are cryogenic milling, wet milling and high-pressure homogenizer and high shear [67]. Lu et al. reported that reduced the size of native starch granules by milling may induce the reduced sizes of starch granules dramatically while damaged their crystalline structure largely [68]. In the research of Villamonte et al., the small starch particles obtained by the high pressure and homogenization process could stabilize the Pickering emulsions [69].

Acid hydrolysis, a chemical method, can reduce the size of particles which are insoluble in water and most organic solvents due to the presence of crystalline structures [67]. Strong acids, such as H_2_SO_4_ and HCL, can remove the susceptible amorphous regions of materials and retain the crystal structure. It has indicated that the acid hydrolysis adding ultrasound can produce starch nanocrystals [70].

#### 3.1.2. Anti-Solvent Precipitation Method

Anti-solvent precipitation method has several advantages such as low cost and easy operation, especially suits for water-insoluble food materials. The first step in this technique is to dissolve the material in a good solvent. And then the solvent with the materials is added to another non-solvent, which is usually an aqueous phase, under stirring conditions with or without the existence of stabilizer [67]. Finally, use a rotary evaporator to remove organic solvent dispersed in an aqueous phase under reduced pressure, and then the materials quickly reach saturation and crystallized out forming nana/micro particles. The premise of this preparation technique is that the solubility of the material molecules in the good solvent and the anti-solvent must have a certain gap, and the good solvent and the anti-solvent can be mutually soluble. In the research of Xiao et al., through anti-solvent precipitation method, they manufactured kafirin nanoparticles, which is the major storage protein from sorghum grain and is soluble in acetic acid or alcohol aqueous solution but insoluble in water [71].

#### 3.1.3. Methods for Preparing Gel Particles

The preparation methods of gel particles are generally divided into two categories: Firstly, mechanical preparation methods, that is, first make a gel, and then pass the extrusion method, spray drying method, shear method and high-pressure homogenization method and other mechanical methods to separate and crush it to obtain micron or nanoscale gel particles. Secondly, molecular association, that is, first make a colloidal solution through the physical and chemical effects, such as thermal denaturation of proteins, chemical crosslinking of polysaccharides and proteins. And then the solute in the solution is made into a gel particle state through re-coagulation, shearing, and homogenization, etc.

The gel particles commonly used to stabilize Pickering emulsions include protein gel particles and protein-polysaccharide complex gel particles, which are connected to each other through covalent bonds to form a spatial network polymer under certain conditions. Protein gel particles are amphiphilic and have surface activity at the interface between oil and water. Guo et al. reported that soy protein based microgel particles could be fabricated through hydrogel preparation and homogenization [72]. Herein, the microgels adsorbed at the interface with strong energy could be used as stabilizers for O/W Pickering emulsions. After the protein and polysaccharide molecule are combined, the electrostatic force can be used to stabilize the colloidal particles, which can be used as emulsifier for Pickering emulsions. It was proposed by Wang et al. that a new edible Pickering emulsion stabilized by zein/chitosan composite particles could be prepared to enhance the oxidative stability of the emulsion [73].

### 3.2. Preparation of Pickering Emulsions

#### 3.2.1. Rotor-Stator Homogenization

The common method for preparing Pickering emulsion is the rotor-stator homogenization method, whose homogenizer is composed of a rotor and a stator [8]. The fixed part of the motor is called the stator, on which a pair of DC-excited stationary main poles is installed. The rotating part is called the armature core, and the armature winding is installed on it. When the bladed rotor rotates, the liquid can be circulated in and out of the container. Due to the acceleration caused by the high-speed rotation of the liquid in the device and the shear force between the rotor and the stator, the emulsion droplet size is reduced. Besides, to reduce the average particle size of homogeneous products, the gap between stator and rotor should be reduced. Emulsion droplet size is affected by homogenization time and rotation speed, which is the numbers of rotations per minute.

Rotor-stator homogenization has many advantages. Firstly, it is easy and convenient to be operated and the cost is low. Secondly, the emulsification process, which just needs a few minutes, is rapid and it is also more energy efficient. In addition, because the rotor-stator is flexible and diverse in design, the technology is widely adaptable and can currently be applied to the market. However, there are some disadvantages here. Emulsified samples may lack uniformity. If the gap between the stator and the rotor is too narrow, it will bring difficulties in component design, processing accuracy and assembly process. When the rotor rotates at high speed, the rotor is likely to collide with the stator after deformation. Additionally, during the emulsification process, the shear rate between the rotor and the stator is high, which can damage or deform fragile particles or aggregates [8].

#### 3.2.2. High-Pressure Homogenization and Ultrasonic Emulsification

**Figure 3:**
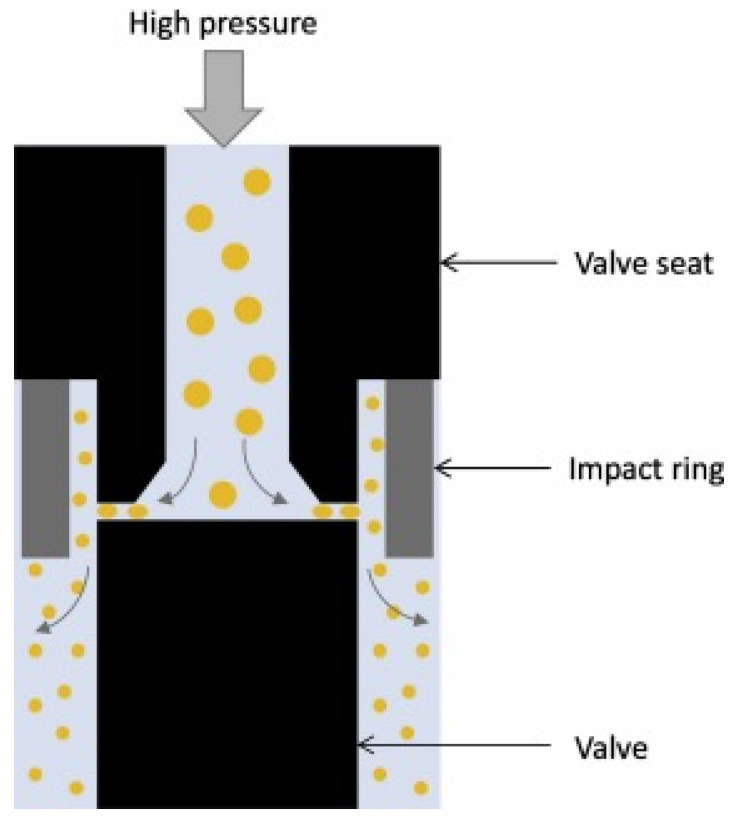
The high-pressure homogenizer with a standard homogenizing nozzle [8].

The high-pressure homogenization method is a more commonly used continuous emulsification process for preparing Pickering emulsions. Its main equipment includes high-pressure pumps and homogeneous nozzles as shown in Figure 3. The first step is pre-emulsification to obtain a coarse emulsion, and then pass the primary emulsion through the slits of the high-pressure homogenizer, and rely on its cavitation, turbulence and shear to prepare the primary emulsion into a fine emulsion [74,75]. A large amount of data shows that the droplets formed by high-pressure homogenization are smaller than those formed by the rotor-stator homogenization [8]. High-pressure homogenization has many advantages, such as the ability of the equipment to produce continuously and the possibility of obtaining small and uniform droplets.

Ultrasound uses a frequency above 16k Hz, which can interact with substances and be used for emulsification [8]. The ultrasonic method also uses cavitation, turbulence and shear stress to prepare the emulsion, which can make the stabilizer adsorb on the two-phase interface. Herein, cavitation causes local high temperature, high pressure and stress during the formation of the emulsion, which is beneficial to the formation of Pickering emulsion. The most commonly used equipment for the preparation of Pickering emulsion is an ultrasonic probe. It can transmit ultrasonic energy to surrounding samples and thus inducing emulsification. It has been demonstrated by many researches that the droplet size of the Pickering emulsion is close to that obtained by high pressure homogenization.

However, these two methods also have certain limitations. Because the emulsion is in a high shear state during preparation, it is easy to destroy the agglomerates of its stabilizer, which leads to the aggregation and stability of the emulsion. In addition, due to the local high pressure and high temperature, the energy consumption is large during the preparation process resulting in the high operating cost.

#### 3.2.3. Membrane Emulsification

**Figure 4:**
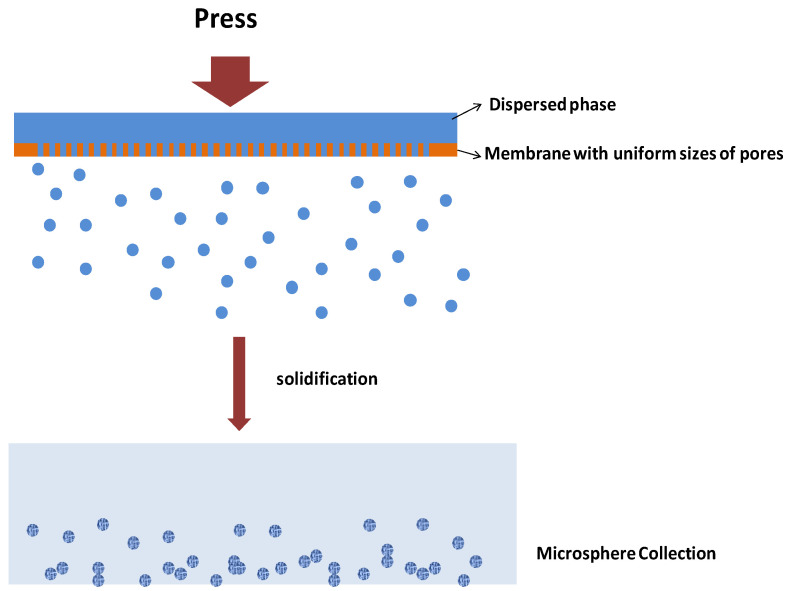
Schematic description of processes of direct membrane emulsification method.

**Figure 5:**
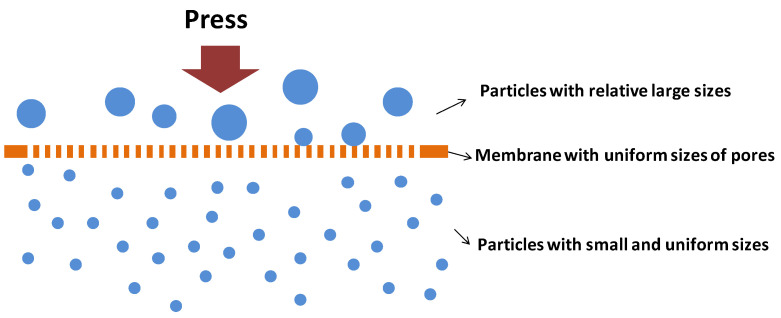
Schematic description for the process of rapid membrane emulsification.

Membrane emulsification technology refers to a method of pressing pure dispersed phase or primary emulsion into a microporous membrane and controlling the injection rate and shearing conditions to prepare a Pickering emulsion. The two governing classes of membrane emulsification technology are direct membrane emulsification (DME) and premixed membrane emulsification (PME) as shown in Figure 4 and Figure 5 [8]. Apparently, the pore size of the membrane, the viscosity of the continuous phase and the dispersed phase and the size of the surface tension are important factors affecting the droplet size of the Pickering emulsion. Manga et al. showed that emulsion droplets could be produced through rotary membrane emulsification and using silica colloids as a sole stabilizer [76]. Compared with conventional preparation methods, Membrane emulsification, an environmentally friendly method with application potential, requires less energy to prepare an emulsion of the same particle size, and the particle size of the emulsion is uniform. However, it also has some disadvantages. The process needs a long time and only suitable for low viscosity systems. So the limitation of this method is relatively low in emulsification yield, and there are currently few studies on this method.

#### 3.2.4. Microfluidic Technology

**Figure 6:**
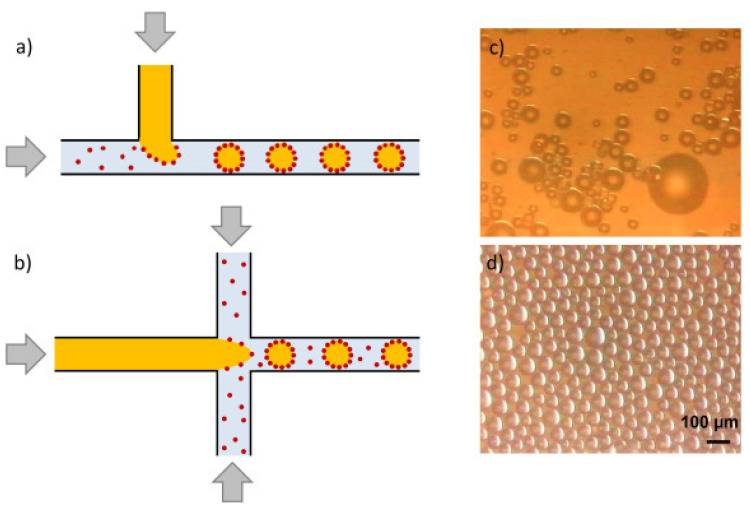
Microfluidic devices (**a**) T-junction and (**b**) flow-focusing for Pickering emulsions preparation. Light micrograph images of silica-stabilized emulsions by (**c**) a homogenizer and (**d**) a microchannel emulsification [8].

Microfluidic technology, a drop-by-drop technology, is also a new method of preparing emulsion in recent years [77]. The principle of emulsion formation in the microfluidic method is that the dispersed phase flows in parallel and the continuous phase flows vertically and the dispersed phase forms spherical droplets under the drag of the continuous phase when they meet as shown in Figure 6. Through microchannel emulsification method, Xu et al. produced O/W Pickering emulsion which was only stabilized by silica particles [78]. Compared with emulsions prepared with conventional homogenizers, the obtained emulsions have greatly superior stability. The microfluidic technology is a gentle and promising method without high shear forces, so it does not destroy the agglomerates of the stabilizer, thereby forming a thick film around the droplets to stabilize the emulsion. Additionally, Microfluidic method may offer several advantages in simple preparation and precise control of emulsion droplets.

## 4. Stability Mechanism, Degradation Mechanism, and Stability Control Method of Pickering Emulsions

### 4.1. Stability Mechanism of Pickering Emulsions

#### 4.1.1. Theory of Solid Particle Interface Film

**Figure 7:**
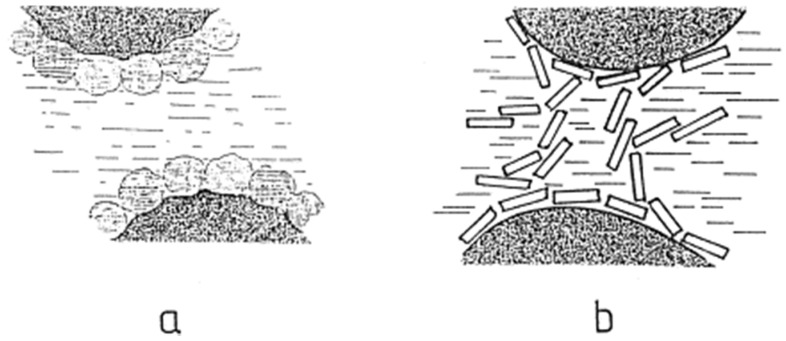
Stabilization mechanisms of the Pickering emulsion. (**a**) Stabilized by envelopes of particles around the oil droplets; (**b**) stabilized by encapsulation of oil droplets in a three-dimensional network of particles [85].

The theory of solid particle interface film means that due to the capillary force, the partially wetted solid particles in two phases can be adsorbed on the interface to form a dense single-layer or multi-layer particle film as shown in Figure 7a. The particle film is a physical barrier that can prevent droplets from touching and aggregating each other, which increases steric hindrance and improves the rheology and shear properties of the interface film [79,80,81]. The desorption energy of the solid particles at the oil–water interface is much greater than the thermal energy, so the adsorption of particles is irreversible [82,83]. In addition, since the solid particles in water adsorbed on the droplet surface have surface charges, the mutual repulsion between the droplets is enhanced [84].

#### 4.1.2. Three-Dimensional Viscoelastic Particle Network Mechanism

The three-dimensional viscoelastic particle network mechanism refers to the formation of a three-dimensional network structure between particles and particles, and between particles and droplets under van der Waals force, which is also an important factor to stabilize the Pickering emulsions in Figure 7b. Lagaly et al. proposed a three-dimensional viscoelastic particle network mechanism capable of stabilizing Pickering emulsions in the study of smectites as a colloidal stabilizer for emulsions [85]. Apparently, studies have shown that this structure can increase the viscosity of the emulsion, reduce the migration rate of particles and reduce the rate of merger of droplets, thereby slowing the delamination of the emulsion and preventing aggregation of droplets [84,86]. The wettability, concentration of solid particles and electrolyte concentration, the pH value of the water phase all have effects on the stability of Pickering emulsions.

### 4.2. Degradation Mechanism of Pickering Emulsions

The emulsion system is a thermodynamically unstable system, so when affected by some external conditions (such as temperature, pH, electrolyte concentration, particle concentration, storage time, etc.), the emulsion will degrade. In food applications, researchers add emulsifiers so that the emulsion is not destroyed before the shelf life of the product [2,87]. However, the degradation trend of the emulsion is inevitable, and it is just a matter of time.

#### 4.2.1. Physical Degradation

It is well known that the free energy formula of emulsion system is ΔG = γ ΔA, where γ is the tension of the oil–water interface and ΔA is the total interface area of the system [7]. Usually researchers add surfactants or solid particles to adsorb at the oil–water interface to reduce the interfacial tension, thereby reducing the free energy of the system to slow down the emulsion degradation. However, the free energy ΔG is positive, so the emulsion will eventually show a phase separation [7].

The most common physical degradation is gravity settling due to the density differences between the continuous phase and the dispersed phase [7]. The emulsion will show creaming and sediment due to the differences in density of the two phases. With the density differences between the continuous phase and the dispersed phase becomes smaller, the droplet radius will become smaller, the viscosity will become larger, the gravity sedimentation rate will become slower and the emulsion will be more stable. Emulsion droplet flocculation means that two or more droplets attract each other to form flocs, but it has been found through research that the droplets maintain their inherent properties and this phenomenon is reversible. Therefore, the stability of the emulsion can be optimized by adjusting the wettability and concentration of the Pickering emulsion particles. The effect of flocculation on the emulsion is complex, and a lower degree of flocculation can increase the viscosity of the system, thereby improving the stability of the emulsion. The third type of degradation is droplet coalescence, that is, two droplets collide with each other, causing water to flow out from the space between the droplets, and the interface film becomes thin and breaks [88]. Contrary to flocculation, after the interface membrane ruptures, the droplets merge with each other and cannot maintain their original properties. This process is irreversible. Ostwald ripening is another degradation phenomenon, that is, due to the difference in internal pressure between droplets of different sizes, droplets with small size will be transferred to droplets with large size resulting in emulsion aggregation, and eventually leading to a macroscopic stratification [88,89]. Due to the irreversible adsorption of particles at the interface and the steric hindrance formed by the particles at the interface, Pickering emulsions hardly undergo Ostwald ripening. The last one is the phase change of the emulsion, that is, due to changes in some environmental factors (such as temperature, ion strength, pH), which cause some changes in the viscosity of the system, the continuous phase and the dispersed phase switch between each other [90].

#### 4.2.2. Chemical Degradation

Because the Pickering emulsion is composed of an oil phase and an aqueous phase, the system is prone to degrade due to the oxidation of polyunsaturated fatty acids. In addition, during the processing of food-grade Pickering emulsion, due to environmental changes, it will also lead to lipid oxidation and degradation of some biologically active substances, which will change the flavor and quality of food. It reported that the interface composition and properties of the system was the key to inhibit lipid oxidation, and the chemical degradation can be slowed by the steric hindrance of the interface membrane. Biopolymer-based particles of flaxseed protein and polysaccharides were used to stabilize the Pickering emulsion of flaxseed oil, whose oxidation stability was increased, and the addition of thymol to the oil phase further depressed the oxidation of flaxseed oil [91]. Hosseini et al. reported that a flaxseed oil (FSO)-in-water Pickering emulsion could be stabilized by chitosan-myristic acid (CS-MA) nanogels and clove essential oil (CEO), which was around dispersed droplet surfaces [92]. Herein, steric hindrance of CS-MA nanogels and the interfacial load of CEO could inhibit the oxidation of the emulsion.

### 4.3. Stability Control Methods

#### 4.3.1. Particles Modification

The wettability of solid particles is a key factor in the stability of Pickering emulsions and is generally characterized by the three-phase contact angle (θ). Studies have shown that when θ approaches 90°, the stability of the emulsion is the largest. When θ approaches 0° or 180°, the particles have too strong hydrophilicity or lipophilicity to form the emulsion with improved stability. Therefore, we modify the wettability of particles through particle modification [17,93,94].

Firstly, we use physical adsorption to adsorb small molecules and polymers to the surface of particles by non-covalent bonding, and then adjust surface charge and hydrophobicity of the particles to optimize the wettability of the particles in two phases [93,95]. Generally, a larger electrostatic repulsion is formed between high-charge particles, which can effectively prevent the collision and aggregation of oil droplets. But when it is too high, the particles cannot be well adsorbed at the interface, resulting in emulsion delamination. In addition, the hydrophobic particles in the middle have better wetting in the two phases [17]. Binks et al. reported that inherently hydrophilic nanoparticles could form hydrophobization in situ by adsorbing dissolved oil molecules of dialkyl adipate oils to obtain Pickering emulsions with desired stability [96]. The second method is chemical anchoring, which fixes specific groups on the surface of particles by the action of covalent bonds to adjust the wettability of particles [93]. It was proposed by Saigal et al. that silica nanoparticles with thermally responsive polymers grafted from their surfaces could stabilize Pickering emulsions [97]. In addition, SannaBjörkegren et al. used hydrophobic propyl and methyl silanes and hydrophilic mPEG silane to adsorb on the silica particle surface improving emulsification property and stability of emulsions [98].

#### 4.3.2. Adjustment of Particles Concentration

Particle concentration has an important effect on emulsions stability and droplet size. When the particle concentration is low, the size of the emulsion droplet is the largest, and the emulsion is the most unstable, which is prone to flocculation. When the particle concentration is high, a viscoelastic network structure can be formed between the particles, and the droplet size is also reduced, forming a relative stable emulsion system. When the particle concentration further increases, the droplet size decreases to a certain extent and does not change. As the concentration of zein-propylene glycol alginate particles increased from 0.25% to 2.00%, the diameter of the emulsion decreased from about 90 μm to 19 μm [42]. In addition, they found that as the concentration of zein-propylene glycol alginate particles (ZPGAPS) increased, the emulsion stability of the emulsion increased significantly.

#### 4.3.3. Others

pH can change the interface charge of Pickering emulsion, which can adjust the surface wettability and interfacial tension of particles, and thus change the adsorption capacity of particles at the interface. When the pH value reached the isoelectric point, the electrostatic repulsion between the particles is weak, and the wettability in two phases becomes smaller, the emulsion particle size increases, and the emulsion become unstable. Xiao et al. used grafted carboxymethyl starch nanoparticles to stabilize pH-responsive Pickering emulsion. Herein, the Pickering emulsion had highly viscous and strong stability at pH 6.0 and 10.0 [99]. However, due to the weak charge of starch, emulsion droplets exhibited flocculation at low pH value near its isoelectric point.

The electrolyte concentration also affects the stability of the emulsion by regulating the particle interface charge. By adjusting the ionic strength of the system, the degree of flocculation between the droplets and the interface adsorption behavior of the colloidal particles can be adjusted to control the stability of the emulsion. It is known that when the ion concentration is low, the particles adsorbed on the interface increase, which will produce appropriate flocculation and enhance the protection of the interface film. In another case, when the ionic strength is too high, the particle size is too large falling off from the interface film and the stability of the emulsion is greatly reduced. Anjali et al. showed that the electrolyte shielded the surface charge of particles and accelerated the adsorption of the particles to the interface possessing the emulsion with improved stability at a moderate salt concentration [100].

Temperature has an important influence on the preparation and storage of Pickering emulsion. We should look for the optimum temperature for solid particles and emulsions. In addition, different temperatures will produce emulsions with different properties. The oil–water volume ratio is another important factor to affect the type and stability of the emulsion. The change in oil–water ratio will cause two phases of the emulsion to reverse. When minimal internal phase ratio is from 0.75 to 0.85, a high internal phase Pickering emulsion with improved stability with starch nanocrystals can be formed [101].

## 5. Applications of Pickering Emulsion in Food Field

Currently, as obesity, urbanization, and population aging becoming key global challenges, the growing demand for food safety and healthy forces food researchers to explore possibilities in more areas to address the unresolved issues in food design challenge. Pickering emulsion has a wide range of applications in the food industry. The following is a summary of various food applications of Pickering emulsion in the past three years.

### 5.1. Dairy Products

Dairy products include liquid products (such as pasteurized milk, sterilized milk, yogurt), solid dairy products (such as cheese, fresh cream, ice cream, etc.). These dairy products were unstable because of the influence of external conditions during the production and storage process, which may lead to changes in the flavor and quality of the food. Therefore, we need to add stabilizers or emulsifiers to stabilize the emulsion system. The milk in pasteurized milk or sterilized milk contains 4% milk fat (equivalent to the dispersed phase in the emulsion), which can form tiny balls and disperse well into the emulsion being an emulsified state. It also contains protein, of which 80% is casein and 12% is whey protein. Casein is heat-resistant and can combine with calcium and phosphorus to form casein particles, which are suspended in the milk with colloidal suspension and act as a surfactant. Due to fine size of casein particles, it can keep the natural milk emulsion with improved stability physically. Additionally, casein micelles are functional colloids that can be considered as natural sophisticated delivery systems for micronutrients, such as calcium and phosphate [5]. But under acidic conditions, casein will precipitate. Whey protein is sensitive to heat, and it will change quality and precipitate during heating. We can change the heat resistance of whey protein by ultrasonic treatment or synergy with some substances [102,103]. Additionally, through Maillard reaction, which links the amino groups of protein and carbonyl groups of polysaccharides, the physiochemical properties of whey protein are improved [104,105]. In the research of Liu et al., WPI-Lac/EGCG nanoparticles could keep the emulsion with desired stability in a water bath at 85 °C for 30 min [106]. Compared with WPI, the saccharified WPI-Lac/EGCG nanoparticles stabilized emulsion showed more uniform droplet distribution, stronger thermal stability and higher curcumin retention rate.

### 5.2. Encapsulation and Controlled Release of Functional Food Ingredients

**Figure 8:**
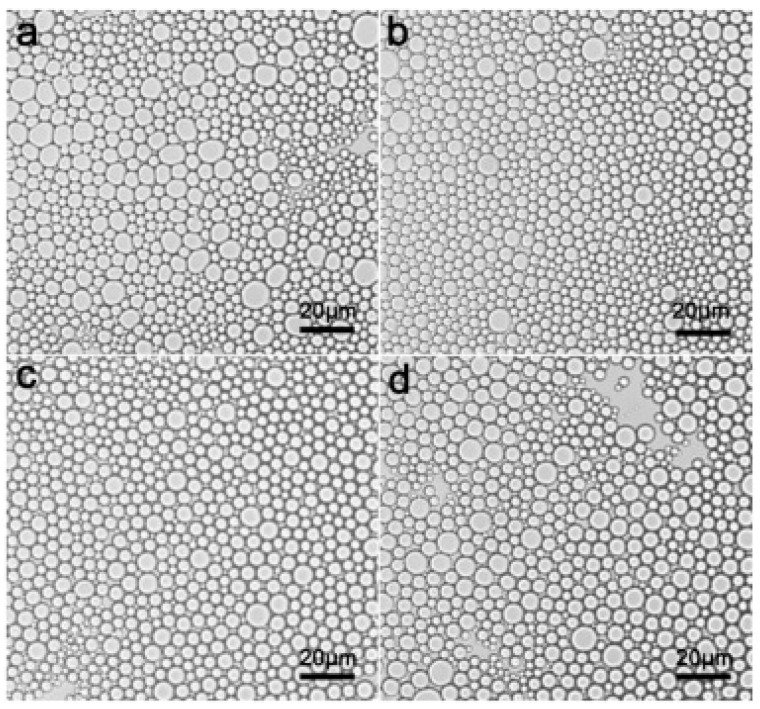
Micrograph images of emulsions stabilized by (**a**) IDF(water-insoluble dietary-fibers )-0, (**b**) IDF-10, (**c**) IDF-30, (**d**) IDF-50 dispersions respectively, the solid content of the IDF dispersions was 0.3 wt%, and the volume ratio of oil to water was 3:7 [117].

Functional food refers to food containing some special functional ingredients and active ingredients such as vitamins, dietary fiber, oligosaccharides, protein and bioactive substances. It can regulate body functions and prevent diseases, such as anticancer and anti-inflammatory capacities [17,107,108]. However, these ingredients may not be easily soluble in water and unstable in the external environment, so we can use the characteristics of Pickering emulsion to encapsulate these active ingredients to improve the process stability and bio-accessibility for targeted delivery and controlled release, and then they can be effectively absorbed by the body. Recently, increasing research has found that complexation between oppositely charged biopolymers may be beneficial to construct active ingredients delivery systems, which has better biosafety and biocompatibility [109,110,111]. In recent years, there are several researches regarding complexes made of proteins with opposite charges [112]. Wei et al. used hetero-protein complex formation of ovotransferrin and lysozyme as food-grade Pickering emulsifier, which has good biocompatibility and can reduce iron-deficiency and anemia. Herein, the complex particles could convey curcumin increasing its bioaccessibility from 16.1% to 38.3% and improving extent of lipolysis [113]. Su et al. used β-lactoglobulin-gum arabic particles with a core-shell structure to stabilize lutein Pickering emulsion, which can protect the retina of human eyes. Herein, lutein is a natural nutrient, but has low water-solubility and poor chemical stability [114,115,116]. Additionally, some active ingredients can be modified by themselves to form a Pickering emulsion with improved stability. He et al. obtained water-insoluble dietary-fibers (IDF) from flammulina velutiper, which have anti-tumor, anti-oxidation, anti-fatigue activities, with high pressure homogenization and then used them as solid particles to stabilize the O/W Pickering emulsion. In Figure 8, we can find that when using a high-pressure homogenization after 10 cycles at 700 bar, the size of emulsion droplets is the smallest and the Pickering emulsion is with the most desired stability in that condition [117,118].

### 5.3. Food-Grade Materials

Food-grade Pickering emulsion via polymerization can synthesize polymers, such as nanocomposites for potential food packaging applications. Some studies used starch nanocrystal stabilized Pickering emulsions by one step polymerization to obtain the production of starch nanocrystal nanocomposite, which was used to produce films with better optical and mechanical quality [17]. Actually, biodegradable packaging films can extend the shelf life of food. Dammak et al. used Pickering emulsions encapsulated hesperidin and stabilized by chitosan nanoparticles obtaining gelatin-based films with good flexibility and strong antioxidant activity [119]. Additionally, Almasi et al. fabricated an active pectin film with excellent mechanical properties and waterproof abilities by majoram essential oil-loaded Pickering emulsions, which were stabilized by WPI–inulin complexes [120].

Several studies show that biological-particle-stabilized Pickering HIPEs with favorable biosecurity and biocompatibility can be used to prepare functional materials, such as highly porous materials, which possess excellent application prospects in food oil sorption, membrane processes, packaging, separation science, and so on [121]. Currently, food-grade particles, including gliadin–chitosan hybrid particles, cellulose nanocrystals and gelatin particles, have been used to stabilize HIPEs. Zhou et al. used gliadin–chitosan complex particles (GCCPs) obtaining Pickering HIPEs with internal phases as high as 90%, which promote the development of porous materials with pore structure [121]. Herein, GCCPs around the droplets can form the three-dimensional network to provide interfacial mechanical barriers against coalescence. In addition, the PM-HIPEs with high porosity and multiscale pore sizes can be used as oil absorbents and showed remarkable absorption capacity. Certainly, these Pickering HIPEs and PM-HIPEs obtaining from food-grade materials have a bright future in food applications.

### 5.4. Fat Substitute

Nowadays, obesity is a health problem faced by the whole world, which promotes the requirements for the health and safety of food. As is known to all, excessive ingestion of trans fats (TFs), which are derived from partially hydrogenated oils (PHOs) in processed foods, can cause high risk of cardiovascular diseases [122]. Without PHOs, preparation of edible vegetable oils into solid fats has become a technical challenge for food manufactures. High internal phase emulsions (HIPEs), a promising alternative approach, can alternate liquid oils into solid-like facts to produce substitutes to PHOs [123]. Additionally, crystalline triacylglycerol (TAG) hardstocks, which is the most ordinary way to form liquid oil, contain high amounts of saturated fatty acids (SAFA) increasing the risk of cardiovascular disease. A large number of studies have shown that Pickering emulsions can be used instead of crystalline triacylglycerol (TAG) hardstocks to reduce fat intake and benefiting consumers’ health. Several edible materials such as cellulose derivatives, plant proteins and PSMs have been exploited to fabricate structured edible oils. [118] Jose et al. used cellulose microfibers (CMF) produced from cotton cellulose linters by a high-pressure homogenizer preparing O/W Pickering emulsions, which were considered as an alternative to trans and saturated fats in the food industry [33].

### 5.5. Other Aspects

Pickering emulsion can also be used in soups, sauces, gluten free rice bread and other products, where Pickering emulsion can substitute some ingredients of these products, to improve food quality and maintain food shelf life [17,124]. Pickering emulsion can also be used to prepare various sensitive materials such as temperature, pH, ion, electric, and magnetic, and these materials have potential to be applied in food science [99,125]. In addition, Pickering emulsion system can keep hydrophobic health-promoting agents (such as vitamins, minerals, essential fatty acids, and phytochemicals) with good stability in clear beverages [5].

## 6. Challenges and Prospects

Compared with other reported conventional emulsions, Pickering emulsions are characterized by containing solid particles which can stabilize its structure. Food grade solid particles have a high safety factor, and the application of inorganic materials in food has been limited. In addition, nanotechnology is in the development stage. The toxic side effects of the prepared nanoparticles on the human body and whether it can be biodegraded need to be studied. Generally prepared solid particles do not have good wettability in the oil phase and water phase, and need to be modified by complicated physical adsorption or chemical grafting to improve the wettability of the particles [126]. Pickering emulsions stabilized by a single particle are not very stable, and over time, the degradation of the emulsion will occur. The introduction of composite particles can improve with it. However, these particles increase the difficulty and complexity of preparing Pickering emulsions. Most of research works are still in bench with no comprehensive understanding of Pickering emulsion formation and mechanism of action [11]. In addition, membrane emulsification and microfluidic methods are efficient methods for preparing emulsions, but the scaling-up of Pickering emulsion still needs to be further investigated. With the development of preparation technologies and nanotechnology, the application prospect of Pickering emulsion will be more popular and extensive in food fields. The preparation of composite particles has also become a development trend, and the stability of Pickering emulsion will be further improved [42,127].

## 7. Conclusions

Pickering emulsion is a novel emulsion system that uses solid particles as a stabilizer to form a dense interfacial film. It can prevent the aggregation and precipitation of droplets by increasing steric hindrance, and finally achieve the effect of stabilizing the emulsion. The two most common types are W/O and O/W Pickering emulsions. Compared with conventional emulsions, Pickering emulsion has several advantages such as safety, good stability, environmental friendliness, and good biocompatibility. For this reason, it is being more-and-more widely used in the food industry [75,128]. This article describes food-grade solid particles, including polysaccharides, proteins, and inorganic particles. Most of these particles do not have good dual wettability, so physical and chemical methods are needed to modify these materials. In addition, this article introduces two stabilization mechanisms of Pickering emulsions, which are solid particle interface film and three-dimensional viscoelastic particle network, and some degradation phenomena. Factors affecting the stability of the emulsion are the wettability of solid particles, the concentration of solid particles, the pH value, the electrolyte concentration, and the volume ratio of oil to water. In summary, it has potential to be widely used in dairy products, beverages, condiments and other food fields. In addition, due to people’s emphasis on food safety and health, the application of Pickering emulsion in functional food has also become a research hotspot [9].
